# Functional Motor Assessment and Rehabilitation in Joubert Syndrome: A Narrative Review and Conceptual Framework for Pediatric Neurorehabilitation

**DOI:** 10.3390/children13040512

**Published:** 2026-04-07

**Authors:** Łukasz Mański, Aleksandra Moluszys, Jolanta Wierzba

**Affiliations:** 1 Gdansk Medical Academy of Applied Sciences, 80-335 Gdansk, Poland; aleksandra.moluszys@gam.edu.pl; 2 Department of Internal and Pediatric Nursing, Institute of Nursing and Midwifery, Medical University of Gdansk, 80-208 Gdansk, Poland

**Keywords:** Joubert syndrome, pediatric physiotherapy, neurorehabilitation, motor control, functional assessment, sensorimotor integration, cerebellar disorders

## Abstract

**Highlights:**

**What are the main findings?**

• Available evidence on physiotherapy in Joubert syndrome is limited to low-level studies and shows high heterogeneity in interventions and outcomes;
• Commonly used pediatric outcome measures (e.g., GMFM-88, WeeFIM) may not capture the multidimensional motor phenotype, particularly regulatory and sensorimotor domains.

**What are the implications of the main findings?**

• A preliminary, hypothesis-generating conceptual framework is proposed to support multidimensional functional assessment and clinical reasoning in JS;
• There is a critical need for more sensitive, syndrome-specific assessment tools and higher-quality rehabilitation studies in this population.

**Abstract:**

**Background/Objectives**: Joubert syndrome (JS) is a rare neurodevelopmental disorder characterized by cerebellar and brainstem malformations, resulting in a complex and heterogeneous motor phenotype. Despite increasing clinical recognition, functional assessment and physiotherapy strategies in this population remain insufficiently characterized. This study aimed to synthesize current rehabilitation evidence and to propose a conceptual framework for functional motor assessment in children with JS. **Methods**: A structured narrative review was conducted across PubMed, Scopus, Web of Science, EBSCOhost, the Cochrane Library, and PEDro databases, including studies published between 2000 and 2026. Eligible studies involved pediatric patients (0–18 years) with JS and reported physiotherapy or motor-related outcomes. Data were synthesized descriptively, and recurring functional domains were identified to inform the development of a conceptual framework. **Results**: Ten studies (eight case reports and two case series) were included. Rehabilitation approaches were heterogeneous and predominantly multidisciplinary, focusing on postural control, trunk stability, and motor milestone acquisition. Functional improvements were reported across studies; however, outcome measures were primarily based on generic pediatric tools such as GMFM-88 and WeeFIM. These tools did not fully capture the multidimensional nature of motor impairment, particularly in relation to regulatory and sensorimotor domains. Evidence also suggested that postural control and gross motor performance may not fully correspond, highlighting additional functional components such as axial control and thoracoabdominal organization. Given the absence of formal risk-of-bias assessment and the low methodological quality of included studies, all findings should be interpreted as exploratory. **Conclusions**: Current functional assessment in JS may not adequately reflect the interaction between regulatory processes, sensorimotor integration, and motor control. The proposed conceptual framework provides a multidimensional, hypothesis-generating perspective that may support clinical reasoning and physiotherapy planning. Further research is required to validate this framework and to develop more sensitive, syndrome-specific assessment tools.

## 1. Introduction

Joubert syndrome (JS) is a rare neurodevelopmental disorder characterized by congenital malformation of the cerebellar vermis and brainstem [1,2]. The condition is typically identified by the characteristic “molar tooth sign” visible on axial magnetic resonance imaging (MRI), reflecting abnormalities of the superior cerebellar peduncles and the deep interpeduncular fossa [3]. Clinically, JS is associated with hypotonia, delayed motor development, abnormal eye movements, and episodic breathing disturbances during infancy [1,3]. Due to its early clinical presentation, JS may initially be misdiagnosed as hypotonic cerebral palsy (CP) before characteristic neuroimaging findings are recognized [4].

The neurological features of JS arise primarily from structural abnormalities affecting the cerebellum and brainstem [1,3]. These structures play an important role in the coordination of movement, integration of sensory inputs, and regulation of autonomic functions [5]. Dysfunction within cerebellar–brainstem networks may therefore influence multiple physiological systems involved in motor control [6]. In addition to hypotonia and ataxia, many children with JS present with irregular breathing patterns, oculomotor abnormalities, and disturbances of postural regulation. The disorder is also recognized as a multisystem condition, with possible involvement of renal, hepatic, and visual systems, further contributing to clinical heterogeneity [2].

Motor development is frequently delayed and is often characterized by impaired postural control, balance difficulties, and delayed acquisition of gross motor milestones such as sitting, crawling, and independent walking [1]. Cerebellar dysfunction may further influence movement timing, coordination, and motor planning [7]. Visual and oculomotor disturbances may also affect visuomotor integration and spatial orientation during movement [8]. Together, these factors contribute to a complex motor phenotype that may substantially affect daily functioning and participation.

Although the neurophysiological mechanisms underlying motor control have been extensively described in the motor control and neuroscience literature [9], their translation into clinical assessment frameworks for rare cerebellar–brainstem disorders remains limited. Existing models of postural regulation and sensorimotor control emphasize dynamic interactions between regulatory physiological systems, sensory inputs, motor control processes, and motor output in the organization of movement [9,10]. These theoretical perspectives have been only partially translated into rehabilitation research in JS, where the available literature remains sparse and is largely dominated by case reports and small case series [1].

Most studies rely on generic pediatric developmental scales that were not specifically designed to capture the multidimensional motor characteristics associated with cerebellar–brainstem dysfunction [11]. Consequently, functional domains such as regulatory physiological inputs, cerebellar motor control mechanisms, and sensorimotor integration may be insufficiently represented or entirely absent in commonly used pediatric outcome measures [12].

The aim of this article is twofold: first, to synthesize the available evidence on physiotherapy and rehabilitation in children with JS, and second, to propose a preliminary, hypothesis-generating conceptual framework for functional motor assessment integrating regulatory processes, sensorimotor inputs, and motor performance. This framework is intended to provide a conceptual structure that may support physiotherapy assessment and clinical reasoning during rehabilitation planning in children with JS.

## 2. Materials and Methods

### 2.1. Study Design

This study was conducted as a structured narrative review synthesizing the available rehabilitation literature on JS and identifying functional domains relevant to physiotherapy assessment and intervention. Given the rarity of JS and the limited number of rehabilitation studies, the objective of this review was not to quantitatively evaluate treatment effectiveness but rather to provide a structured overview of reported rehabilitation approaches and functional outcome measures used in this population. In addition to summarizing the available evidence, the review sought to identify recurring functional themes emerging from the literature that could inform the development of a conceptual framework for functional motor assessment in children with JS.

### 2.2. Literature Search

A literature search was conducted across multiple biomedical databases to identify peer-reviewed publications related to rehabilitation in JS. The databases included PubMed, Scopus, Web of Science, EBSCOhost, the Cochrane Library, and PEDro. The search strategy combined keywords related to JS and rehabilitation, including “Joubert syndrome”, “physiotherapy”, “physical therapy”, “rehabilitation”, “motor function”, “motor development”, and “functional assessment”. These terms were combined using Boolean operators, and the search strategy was adapted to the indexing structure of each database. The search included articles published between 1 January 2000 and 15 March 2026 and was limited to English-language publications involving human participants. Only studies addressing pediatric populations aged 0–18 years were considered. All identified records were imported into Zotero(version 8.0.5) reference management software, where duplicate records were removed prior to screening. Reference lists of relevant articles were also screened to identify potentially eligible studies not captured during the database search. The database search identified a total of 1018 records: PubMed (n = 119), Scopus (n = 187), Web of Science (n = 147), EBSCOhost (n = 538), the Cochrane Library (n = 2), and PEDro (n = 25). After removal of duplicates, 421 articles remained for title and abstract screening. Following this stage, 19 full-text articles were assessed for eligibility, of which 10 studies met the inclusion criteria and were included in the qualitative synthesis. The review was conducted in accordance with general principles of narrative synthesis but did not strictly follow formal systematic review guidelines.

### 2.3. Eligibility Criteria

Studies were considered eligible for inclusion if they met the following criteria: (1) involved pediatric patients aged between 0 and 18 years with a diagnosis of JS, (2) reported physiotherapy, rehabilitation, or motor-oriented therapeutic interventions, and (3) included functional, motor, postural, or developmental outcomes relevant to physiotherapy practice. Studies were excluded if they met any of the following conditions: (1) focused exclusively on adult populations, (2) addressed purely genetic, radiological, or surgical aspects of JS without a rehabilitation component, (3) did not report functional or motor-related observations relevant to physiotherapy assessment or intervention, or (4) were conference abstracts without accessible full text.

### 2.4. Data Extraction and Evidence Synthesis

For each eligible study, relevant information was extracted regarding study design, participant characteristics, type of physiotherapy or rehabilitation intervention, duration and frequency of therapy (if reported), outcome measures used and reported functional changes. Because the included studies differed substantially in terms of study design, intervention approaches, and outcome measures, a quantitative synthesis or meta-analysis was not considered appropriate. Instead, findings were synthesized using a descriptive narrative approach. The analysis focused on identifying recurring patterns in rehabilitation strategies and functional outcomes reported in the literature, with particular attention given to functional domains potentially relevant to motor organization in JS. Data extraction and synthesis were primarily conducted by a single author. In cases of uncertainty regarding study eligibility, decisions were discussed with a second researcher to reach consensus. Given the narrative design of this review, the synthesis process is inherently interpretive and may be subject to a degree of subjectivity. No formal standardized critical appraisal tool was applied. Therefore, the interpretability of findings is inherently limited and should be considered exploratory. However, the methodological characteristics of included studies were considered descriptively. All included studies were case reports or small case series, representing low-level evidence with inherent limitations in generalisability and risk of bias.

### 2.5. Development of the Conceptual Framework

Following the synthesis of the available rehabilitation literature, the reported functional outcomes and therapeutic targets were analyzed in relation to the known neurophysiological characteristics of JS, particularly those associated with cerebellar and brainstem dysfunction. Based on this analysis, several functional domains potentially relevant to physiotherapy assessment were identified. These domains were subsequently integrated into a conceptual framework describing key components of motor function organization and potential rehabilitation targets in children with JS. The proposed framework should be considered hypothesis-generating and exploratory in nature, as it is derived from a limited and heterogeneous body of literature. It is intended to provide a preliminary conceptual structure that may support future research and may contribute to the development of more syndrome-specific functional assessment approaches in pediatric neurorehabilitation.

## 3. Results

### 3.1. Current Evidence on Physiotherapy in Joubert Syndrome

Ten studies met the inclusion criteria (Table 1), including eight case reports and two case series, with no randomized or controlled studies identified. Overall, the available evidence on physiotherapy and rehabilitation in children with JS remains limited and is largely derived from observational reports. All included studies were observational in nature (case reports or small case series), with no controlled trials identified.

Most studies described multidisciplinary rehabilitation programmes combining physiotherapy with additional therapeutic interventions. These commonly included occupational therapy, speech and language therapy, developmental stimulation, orthotic management, and educational support [4,17,19]. Rehabilitation was typically initiated during infancy or early childhood following the recognition of hypotonia or delayed motor development. Across the literature, physiotherapy interventions were generally tailored to the individual functional profile of each child rather than delivered according to a standardized protocol. Importantly, physiotherapy in this population extends beyond intervention and includes functional assessment as a key component of clinical practice. However, assessment-focused studies remain scarce. One pilot case series specifically explored multidimensional functional assessment in children with JS, incorporating GMFM-88, BARS, and musculoskeletal and thoracoabdominal measures [21]. This observation further highlights the limited availability of structured assessment approaches tailored to this population.

### 3.2. Reported Rehabilitation Approaches

The included publications describe a range of physiotherapy strategies used to support motor development in children with JS. Most interventions focused on improving postural control, trunk stability, balance, and the acquisition of gross motor milestones. The main rehabilitation approaches identified in the reviewed literature and their therapeutic targets are summarized in Table 2.

Across the included studies, physiotherapy interventions were most directed toward postural control, trunk stability, and the acquisition of motor milestones [15,16,18,22]. In addition, some reports described the use of sensory-oriented and play-based approaches aimed at supporting motor exploration and body awareness [16]. In selected cases, physiotherapy interventions also addressed non-motor domains, particularly respiratory function in children presenting with central breathing dysregulation [17]. Orthotic management and assistive devices were reported in some studies as part of rehabilitation programmes supporting postural alignment and mobility [4,17]. Rehabilitation was typically delivered within a multidisciplinary context and was individualized according to the functional profile of each child. The duration and intensity of interventions varied substantially across reports, ranging from short-term programmes to long-term management extending over several years [14,19]. These observations are based on a descriptive synthesis of the included studies.

### 3.3. Functional Outcome Measures

A variety of outcome measures were used to document functional changes following rehabilitation in children with JS (Table 3), including standardized motor assessments and broader developmental scales.

Across the reviewed studies, outcome measures primarily focused on observable functional performance, such as gross motor abilities, postural control, and functional independence, most assessed using tools such as Gross Motor Function Measure-88 (GMFM-88) and Functional Independence Measure for Children (WeeFIM) [15,18]. Several studies also applied multidomain developmental instruments capturing cognitive, adaptive, and communication functions [13,14,17]. In some cases, assessment was based on clinical observation and longitudinal follow-up rather than standardized tools [19].

Despite this variability, the literature consistently relies on general pediatric assessment instruments rather than condition-specific measures. Notably, one pilot case series reported that postural control outcomes did not consistently parallel gross motor performance, suggesting that axial control and thoracoabdominal organization may represent additional functional domains not fully captured by standard motor scales [21].

### 3.4. Emerging Functional Domains in Joubert Syndrome

Despite heterogeneity in rehabilitation strategies and outcome measures, several recurring functional domains can be identified across the reviewed literature. Postural control and trunk stability emerged as central features, with consistent reports of impaired sitting balance and proximal stability [15,16,18]. Delayed acquisition of gross motor milestones, including sitting, crawling, standing, and independent walking, was also frequently observed [16,20]. In addition, some reports highlighted difficulties related to coordination, sensory processing, and body awareness [16]. Across studies, rehabilitation was generally associated with gradual improvements in motor abilities, particularly in trunk control, sitting balance, and early ambulation [15,18]. However, persistent developmental delays were commonly reported despite ongoing therapy. These domains suggest that motor impairment in JS extends beyond isolated motor deficits and involves multiple interacting functional systems. These observations provide a preliminary, descriptive basis for the development of a hypothesis-generating conceptual framework proposed in the following section.

### 3.5. Methodological Profile of Included Studies

Given the exploratory nature of the available evidence, a simplified overview of study designs and their inherent methodological constraints is presented (Table 4). The included literature consists exclusively of case reports and small case series, representing low-level evidence.

According to the Oxford Centre for Evidence-Based Medicine Levels of Evidence, the included studies correspond to Levels 4–5, reflecting their descriptive and non-comparative design. These study types are inherently associated with important methodological limitations, including lack of control groups, small sample sizes, and increased susceptibility to selection and publication bias. Substantial heterogeneity was observed across studies in terms of patient characteristics, interventions, and outcome measures, further limiting comparability. No formal risk-of-bias or critical appraisal tool was applied; therefore, this assessment is based on a descriptive appraisal of study design characteristics.

Overall, the available evidence base is limited in both quantity and methodological robustness. Accordingly, the findings of this review should be interpreted with caution and considered exploratory and hypothesis-generating rather than confirmatory.

## 4. Limitations of Current Functional Assessment

### 4.1. Reliance on Generic Outcome Measures

Current functional assessment in children with JS relies largely on generic developmental and motor outcome measures that were originally developed for other neurological conditions, particularly cerebral palsy or global developmental delay. Widely used instruments such as the GMFM-88, WeeFIM, and the Gross Motor Function Classification System (GMFCS) primarily evaluate observable functional abilities, including sitting, standing, walking, and activities of daily living. Although these tools provide valuable information regarding overall functional performance and independence, they were not specifically designed to capture the unique motor characteristics associated with cerebellar–brainstem disorders.

### 4.2. Underrepresentation of Cerebellar and Brainstem Domains

JS is characterized by structural abnormalities affecting the cerebellum and brainstem, which play essential roles in the integration of sensory information, coordination of movement, and regulation of autonomic functions [5]. Disturbances within these neural systems may influence several physiological processes relevant to motor organization. These include respiratory rhythm, postural regulation, oculomotor control, and sensorimotor integration [7,23]. Despite the importance of these mechanisms, these domains are rarely systematically assessed in commonly used pediatric motor outcome measures. Cerebellar contributions to movement timing, prediction, and coordination are well-established in neuroscience research but remain difficult to capture using standard clinical outcome measures [12,24]. Similarly, respiratory dysregulation and other brainstem-mediated regulatory functions are typically not included in standard motor assessments used in pediatric rehabilitation [25]. As a result, functional scales primarily capture observable motor outcomes without fully representing the regulatory and sensorimotor processes that contribute to movement organization in children with JS.

### 4.3. Influence of Visual and Cognitive Factors on Assessment

JS is a multisystem disorder in which motor impairment frequently coexists with oculomotor abnormalities, visual impairment, and varying degrees of intellectual disability [1]. Visual disturbances, such as oculomotor apraxia or retinal involvement, may significantly affect visuomotor integration and spatial orientation during movement tasks [26]. Similarly, cognitive limitations may influence the child’s ability to understand instructions, sustain attention, and engage with structured assessment procedures [27].

These factors may directly influence performance during standardized motor testing. In such cases, reduced scores may not solely reflect motor impairment but may also be influenced by visual or cognitive constraints. This introduces a potential source of bias in the interpretation of functional assessment results and highlights the complexity of evaluating motor function in children with JS. These limitations are summarized in Table 5, which outlines functional domains relevant to JS and their representation in commonly used pediatric outcome measures.

### 4.4. Conceptual Gap in Current Assessment

Contemporary models of motor control emphasize that movement emerges from dynamic interactions between regulatory physiological inputs, sensorimotor integration, motor control processes, and functional motor performance [9]. Rather than representing isolated motor outputs, functional motor performance reflects the integration of multiple interacting processes that contribute to the organization of movement [28].

However, these multidimensional interactions are not explicitly represented within current clinical assessment approaches used in pediatric rehabilitation. Most available tools focus primarily on observable motor performance while providing limited insight into the regulatory and sensorimotor processes that may influence motor behavior. Taken together, these limitations highlight a conceptual gap in current functional assessment strategies for children with JS. This gap may limit the ability of clinicians to fully interpret the mechanisms underlying motor performance and variability. Addressing this limitation may require a more integrative perspective that considers regulatory physiological inputs, motor control processes, and functional motor performance within a unified framework for clinical reasoning.

The conceptual framework proposed in the following section aims to provide such a structure for functional motor assessment and rehabilitation planning in pediatric JS.

## 5. Conceptual Framework for Functional Assessment in Joubert Syndrome

Contemporary models of motor control conceptualize movement as an emergent property arising from interactions among neural, sensory, and regulatory systems [29,30]. Motor behavior is generated through dynamic feedback and feedforward mechanisms integrating sensory input, postural regulation, motor planning, and environmental context [30,31]. These processes involve distributed neural networks, including the cerebellum, brainstem, basal ganglia, and cortical motor areas, which interact to coordinate movement and generate adaptive motor output [17,32].

In cerebellar–brainstem disorders such as JS, disturbances within these networks may affect multiple levels of motor organization [1,3]. Structural abnormalities involving the cerebellar vermis and brainstem may disrupt physiological regulation, sensorimotor integration, and movement coordination [1,33]. As a result, motor performance observed during functional tasks may reflect not only peripheral motor capacity but also alterations in underlying regulatory and sensorimotor processes [34,35].

**Figure 1:**
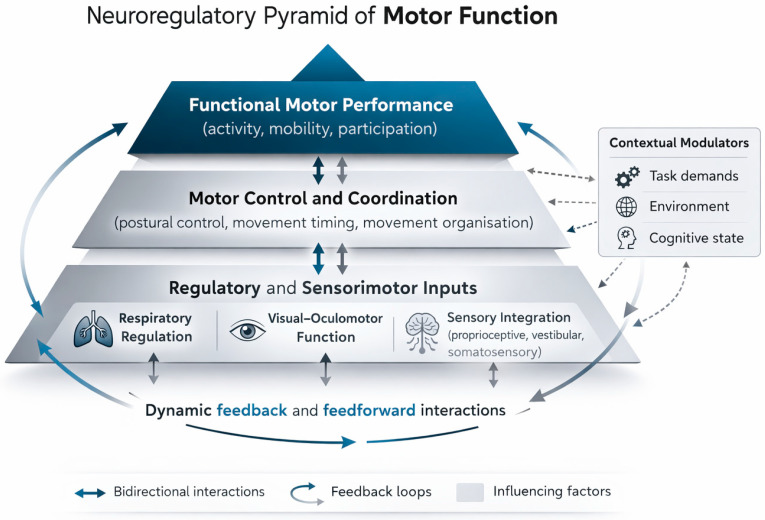
Neuroregulatory Pyramid of Motor Function Conceptual model illustrating interactions between regulatory and sensorimotor inputs, motor control processes, and functional motor performance. Bidirectional connections and circular feedback loops represent dynamic feedforward and feedback mechanisms underlying adaptive motor behavior. The bottom arrow represents a global feedback loop integrating functional motor performance with regulatory and sensorimotor systems, reflecting continuous system-level interactions rather than a single directional pathway.

Based on the synthesis of available rehabilitation literature and current neurobiological knowledge, this study proposes a conceptual framework for functional motor assessment (Figure 1). The model organizes motor function into three interacting levels: (1) regulatory and sensory inputs, (2) motor control processes, and (3) functional motor performance. These levels are not sequential but represent dynamically interacting domains that together shape movement organization [10,31]. The framework is intended as a conceptual tool to support clinical reasoning rather than a mechanistic model of motor control.

Importantly, each level reflects a distinct aspect of motor organization: regulatory and sensory systems provide the physiological and perceptual foundation for movement, motor control processes transform these inputs into coordinated action, and functional motor performance represents the observable outcome of these interactions. The framework complements, rather than replaces, standardized outcome measures by providing a broader perspective on motor organization in children with JS [17,36].

### 5.1. Regulatory and Sensory Inputs

The first level of the framework concerns physiological and sensory systems that influence movement organization. JS is a multisystem neurodevelopmental disorder involving respiratory regulation, visual systems, and sensory processing in addition to motor pathways [1,3]. Structural abnormalities affecting the cerebellum and brainstem may disrupt regulatory mechanisms that contribute to postural control and movement stability [1,33].

Irregular respiratory patterns, particularly in early development, may interact with postural control through respiratory–postural coupling mechanisms involving coordinated activation of the diaphragm and trunk musculature [37,38]. Disruptions in respiratory regulation may therefore influence trunk stability and movement efficiency. Oculomotor abnormalities and visual disturbances may further affect visuomotor integration and spatial orientation during movement [1,3]. Visual input plays a key role in balance, postural alignment, and motor planning [9,32]. Disturbances within visual pathways may therefore increase movement variability and reduce stability [35]. In addition, vestibular, proprioceptive, and somatosensory inputs contribute to body schema formation and coordinated motor behavior [39]. The cerebellum and brainstem integrate multisensory signals to support adaptive motor control and predictive movement adjustments [12]. Alterations in sensory processing may therefore reduce the efficiency of postural responses and impair coordination [40]. Variability in respiratory patterns, visual fixation, sensory responsiveness, and postural organization may therefore influence motor performance and partly explain variability observed during functional assessment in children with JS.

### 5.2. Motor Control Processes

The second level of the framework represents motor control processes that transform regulatory and sensory inputs into coordinated movement. Motor control arises from distributed neural mechanisms integrating sensory feedback, internal predictions, and adaptive motor commands [29,31]. Within these networks, the cerebellum plays a central role in movement timing, coordination, and predictive motor control [34,41]. Structural abnormalities affecting cerebellar and brainstem circuits in JS may disrupt sensorimotor integration and predictive control. These disturbances may alter the timing and scaling of motor responses, leading to impaired coordination and reduced movement stability [3,35]. Clinically, such impairments may manifest as hypotonia, reduced trunk stability, and difficulties with coordinated voluntary movement. Disruption of anticipatory postural adjustments may further affect proximal stability and functional movement organization [34,42]. At this level, assessment focuses on movement quality, coordination, and postural reactions observed during functional tasks. These observations provide insight into underlying motor control processes and complement standardized outcome measures that primarily capture task completion rather than the mechanisms of movement [36].

### 5.3. Functional Motor Performance

The final level represents functional motor performance as the observable outcome of interactions among regulatory, sensorimotor, and motor control processes. Functional motor behavior reflects the ability of the nervous system to translate internal control processes into effective interaction with the environment [29,31]. Children with JS frequently demonstrate delayed acquisition of gross motor milestones, including sitting, standing, and walking [3]. These delays likely reflect the combined effects of cerebellar and brainstem dysfunction on coordination, postural control, and sensorimotor integration. Despite delayed development, gradual acquisition of motor skills suggests the presence of adaptive and compensatory mechanisms [36].

Commonly used outcome measures, such as GMFM-88 and WeeFIM, primarily capture functional performance at the level of task achievement. While these tools are valuable for documenting progress, they provide limited insight into the regulatory and motor control processes underlying movement. From a neurophysiological perspective, similar functional outcomes may be achieved through different neural strategies and compensatory mechanisms [31,40]. Therefore, functional scores interpreted in isolation may not fully reflect the mechanisms underlying motor organization.

Integrating observations from all three levels of regulatory function, sensorimotor processing, and motor control may enhance the interpretation of functional motor performance and support more individualized rehabilitation planning. This perspective highlights the need for multidimensional assessment approaches that extend beyond task-based performance measures. By integrating regulatory inputs, motor control mechanisms, and functional performance, physiotherapists may be better able to adapt rehabilitation programs to the specific functional profile of each child with JS.

## 6. Clinical Implications for Pediatric Physiotherapy

The conceptual framework proposed in this study may support physiotherapists in structuring functional assessment and rehabilitation planning in children with JS. Rather than focusing exclusively on observable motor outcomes such as sitting or walking ability, physiotherapy evaluation may also consider underlying regulatory and sensorimotor processes that may contribute to movement organization. From a clinical perspective, assessment may include observation of respiratory patterns, visual fixation and oculomotor behavior, sensory responsiveness, trunk stability, and movement coordination. These domains may influence motor performance and may help clinicians interpret variability in functional abilities observed during therapy. Considering these interacting systems may also assist in identifying potential therapeutic priorities. For example, disturbances in postural regulation, sensory processing, or visuomotor integration may influence the effectiveness of motor training and may require targeted intervention strategies.

In this context, the proposed framework may provide a conceptual structure that supports clinical reasoning and individualized therapy planning. By integrating regulatory inputs, motor control mechanisms, and functional performance, physiotherapists may be better able to adapt rehabilitation programs to the specific functional profile of each child with JS.

## 7. Discussion

### 7.1. Interpretation of Current Rehabilitation Evidence

The included studies represent low methodological quality and a limited evidence base; therefore, all interpretations should be considered preliminary and interpreted with caution. The available literature provides initial indications that physiotherapy and multidisciplinary rehabilitation may support aspects of functional development in children with JS.

Across the included studies, several recurring patterns can be identified. Rehabilitation approaches most frequently targeted postural control and trunk stability, reflecting a shared emphasis on proximal motor organization. Improvements were most reported in trunk control, sitting balance, and early motor milestone acquisition, suggesting a delayed but progressive developmental trajectory under rehabilitation support. Similar patterns have been described in other pediatric neurological conditions involving cerebellar dysfunction [17,34]. At the same time, substantial variability was observed in intervention strategies, therapy intensity, and duration, limiting direct comparability between studies. The heterogeneity of interventions and outcome measures precludes identification of optimal rehabilitation strategies and limits the ability to generalize findings across studies.

Differences in patient age and functional severity further contributed to this variability. Younger children were more often described in the context of early developmental milestones, whereas older children showed greater emphasis on coordination and functional independence. The diversity of rehabilitation approaches likely reflects the multisystem nature of JS, where motor, sensory, respiratory, and cognitive impairments interact to influence functional outcomes [3].

However, the current evidence base remains extremely limited and is largely derived from case reports and small case series. As a result, clinical practice in JS continues to rely primarily on individualized assessment and therapist-driven decision-making rather than standardized evidence-based protocols [1,33]. These observations should be interpreted as exploratory trends rather than definitive evidence and are not sufficient to support firm conclusions regarding rehabilitation effectiveness.

### 7.2. Limitations of Current Functional Assessment

An important observation emerging from this review concerns the limitations of currently used functional assessment tools. Most studies relied on widely used pediatric motor outcome measures that primarily evaluate observable motor performance. While these instruments provide valuable information on functional abilities, they may not fully capture the multidimensional nature of motor impairment in cerebellar–brainstem disorders.

Motor performance in JS may be influenced not only by motor capacity but also by regulatory, sensory, and cognitive factors. As a result, functional scores may reflect a combination of motor ability and non-motor constraints, complicating interpretation and potentially obscuring the underlying mechanisms of motor impairment [43,44]. In addition, these assessment tools were originally developed for other neurological conditions, such as cerebral palsy and global developmental delay, and may not adequately reflect the specific characteristics of cerebellar–brainstem dysfunction. Motor impairment in JS involves disturbances across multiple interacting systems, including regulatory physiological processes, sensorimotor integration, and motor coordination. Many of these domains, such as respiratory regulation, oculomotor control, and sensorimotor integration, are not directly represented in conventional pediatric motor outcome measures [17,35].

In addition, important psychometric limitations of commonly used outcome measures should be considered. Tools such as the GMFM-88 and WeeFIM may have limitations in construct validity in capturing the multidimensional motor phenotype of Joubert syndrome, as they primarily assess observable performance rather than underlying regulatory and sensorimotor processes. Furthermore, these instruments may demonstrate reduced responsiveness to subtle functional changes and may be susceptible to floor effects in children with more severe motor impairment, potentially limiting their sensitivity in this population. These limitations highlight the need for more sensitive and syndrome-specific assessment tools in this population. These observations should be interpreted with caution, as they are derived from a limited and predominantly low-level evidence base.

### 7.3. Relevance of the Proposed Conceptual Framework

Importantly, the proposed framework should be interpreted as a hypothesis-generating conceptual model rather than an evidence-derived representation of motor function. It is based on a limited and heterogeneous body of literature and is intended to support clinical reasoning and future research rather than to provide definitive mechanistic conclusions.

The conceptual framework proposed in this study represents an exploratory attempt to conceptually address these limitations by integrating regulatory processes, sensorimotor inputs, and motor control mechanisms within a unified perspective. Within this model, functional motor performance is conceptualized as the outcome of interactions among multiple systems rather than as an isolated measure of motor ability [29,30].

In cerebellar–brainstem disorders such as JS, disturbances within these interacting systems may influence multiple aspects of motor organization simultaneously. Structural abnormalities affecting cerebellar and brainstem circuits may alter the integration of sensory feedback, predictive motor control, and autonomic regulation, all of which contribute to coordinated movement. By organizing functional domains into interacting levels, the proposed framework highlights physiological and sensorimotor processes that may influence motor behavior but are not explicitly represented in commonly used outcome measures. For example, domains such as respiratory regulation, visual–oculomotor function, or sensory integration may play an important role in postural stability and coordinated movement but are rarely considered during routine motor assessment [39].

This integrative perspective is also consistent with the biopsychosocial model of functioning described in the International Classification of Functioning, Disability and Health (ICF) [45], which conceptualizes functional performance as the result of interactions between body structures, body functions, activities, and contextual factors. In a similar way, the proposed framework emphasizes that functional motor behavior reflects interactions between regulatory physiological processes, sensorimotor integration, and motor control mechanisms. Importantly, the framework is not intended to replace existing standardized outcome measures. Rather, it provides an additional conceptual perspective that may support clinical reasoning during physiotherapy assessment. Considering regulatory and sensorimotor influences alongside observable motor performance may help clinicians better interpret variability in functional abilities and identify potential therapeutic priorities in children with JS. It should also be acknowledged that parts of the conceptual development are informed by preliminary observations, including the authors’ own prior work. These contributions should be interpreted with caution and considered as exploratory rather than confirmatory evidence. In the absence of formal quality appraisal, the interpretative component of this review should be understood as conceptual and exploratory rather than evidentiary. Accordingly, the framework should be interpreted as a conceptual tool for clinical reasoning rather than as an empirically validated model of motor function.

### 7.4. Future Directions for Research

Given the rarity of JS, future research will likely require collaborative multicenter studies to improve understanding of motor development and rehabilitation outcomes in this population. Larger datasets collected across multiple clinical centers may allow researchers to better characterize the functional motor phenotype associated with cerebellar–brainstem disorders and to identify patterns of developmental progression across different clinical presentations.

Future studies may also explore whether existing motor assessment tools or cerebellar coordination scales can be adapted to better capture functional characteristics associated with cerebellar dysfunction in pediatric populations. Assessment approaches that integrate regulatory physiological factors, sensorimotor processing, and motor coordination may provide a more comprehensive understanding of functional motor performance. Prospective longitudinal studies may be especially valuable for examining how motor function evolves over time in children with JS and how different rehabilitation strategies influence developmental trajectories [36]. Another promising direction involves the development of multidimensional assessment frameworks that combine functional outcome measures with observational analysis of movement quality, postural control, and sensory integration. Such approaches may allow clinicians to better understand the mechanisms underlying motor variability and functional limitations. Ultimately, these efforts may contribute to the development and validation of more syndrome-specific assessment tools that reflect the multidimensional organization of motor behavior in cerebellar–brainstem disorders such as JS.

## 8. Limitations

Several limitations should be considered when interpreting the findings of this review and the proposed conceptual framework. First, the available rehabilitation literature on JS remains extremely limited and is largely dominated by single case reports and small case series. This restricts the ability to draw generalizable conclusions regarding the effectiveness of specific physiotherapy interventions and reflects both the rarity of the condition and the limited availability of structured rehabilitation research in this population. In addition, substantial heterogeneity was observed across the included studies. The reported cases involved children across a wide pediatric age range, from infancy to school age, during which motor development undergoes significant changes. Differences in age may therefore influence both functional trajectories and responsiveness to rehabilitation interventions. Furthermore, variability in clinical presentation, rehabilitation strategies, therapy intensity, duration of treatment, and outcome assessment methods limits direct comparison between studies and reduces the consistency of reported findings. Another limitation concerns the level of detail provided in descriptions of rehabilitation interventions. Many studies reported only general characteristics of physiotherapy programs without sufficient detail regarding therapeutic progression, underlying principles, or dosage parameters. This limits the ability to identify specific components associated with functional improvement and restricts reproducibility in clinical practice. Considerable variability was also observed in the outcome measures used across studies. Most reports relied on generic developmental or motor scales, such as the GMFM-88 or WeeFIM, which were originally developed for other neurological conditions and have not been specifically validated for JS. As discussed in previous sections, these tools may have limited sensitivity to disorder-specific motor impairments associated with cerebellar–brainstem dysfunction and may not adequately capture regulatory or sensorimotor domains relevant to motor organization. In addition, some measures may be susceptible to floor effects in children with severe motor impairment, potentially limiting the detection of subtle but clinically meaningful changes. The nature of the available evidence also introduces methodological limitations. Because most studies describe individual clinical cases, publication bias cannot be excluded, as cases demonstrating favourable developmental outcomes may be more likely to be reported. Furthermore, the included studies were conducted across different healthcare systems, where access to rehabilitation services, intervention intensity, and clinical practices may vary. These contextual differences may further influence reported outcomes and limit comparability across studies. Finally, the present study represents a structured narrative synthesis rather than a formal systematic review with meta-analysis. Although a comprehensive search strategy was applied, the inclusion of English-language publications only may have resulted in the exclusion of relevant studies. In addition, the proposed conceptual framework is based on the synthesis of a limited and heterogeneous body of evidence combined with current neurophysiological knowledge. As such, it should be considered exploratory and hypothesis-generating. The framework has not yet been empirically validated and requires further investigation in clinical populations. Despite these limitations, this study provides a structured synthesis of the available rehabilitation literature and proposes a clinically oriented conceptual perspective that may support future research and clinical reasoning in pediatric neurorehabilitation.

## 9. Conclusions

The available evidence on physiotherapy in children with Joubert syndrome remains limited and is largely based on small observational studies. Current functional assessment relies on generic pediatric outcome measures that may not adequately capture the multidimensional motor phenotype associated with cerebellar–brainstem dysfunction. This review synthesizes the available literature and proposes a preliminary, hypothesis-generating conceptual framework integrating regulatory, sensorimotor, and motor control processes to support clinical reasoning. Given the limited quality and heterogeneity of the evidence, these findings should be interpreted with caution. Importantly, developing sensitive, syndrome-specific functional assessment tools for Joubert syndrome is likely to be essential for improving clinical decision-making, individualising rehabilitation, and enhancing functional outcomes.

## Figures and Tables

**Table 1:** Characteristics of studies investigating physiotherapy and rehabilitation in children with Joubert syndrome.

Study	Country	Design	Participants	Intervention	Duration
Torres et al., 2001 [13]	USA	Case report	Female child with JS (therapy initiated at 10 months of age)	Early developmental intervention with physiotherapy based on the Portage Guide.	Follow-up until 40 months of age; 4 sessions/week
Dekair et al., 2014 [4]	Saudi Arabia	Case report	18-month-old girl with JS	Multidisciplinary rehabilitation with physiotherapy, occupational therapy, speech therapy and orthoses.	4 months
Gagliardi et al., 2015 [14]	Italy	Case report	Female child with JS (TCTN1 mutation)	Multidisciplinary rehabilitation with physiotherapy, visual stimulation, speech therapy, occupational therapy and cognitive training.	Follow-up infancy-9 years; targeted program 30 sessions
İpek et al., 2017 [15]	Turkey	Case report	19-month-old girl with JS	Neurodevelopmental physiotherapy (Bobath concept).	1 h/day, 5 days/week for 13 months
Saiya & Deshpande, 2023 [16]	India	Case report	15-month-old boy with JS	Play-based physiotherapy (NDT, sensory integration, kinesiotaping, treadmill training).	3 sessions/week for 10 months
Mano et al., 2023 [17]	Japan	Case series	3 children with JSRD (3, 11, 13 years old)	Multidisciplinary rehabilitation with physiotherapy, occupational therapy, speech therapy, respiratory therapy and orthotics.	Variable frequency
Devi et al., 2024 [18]	India	Case report	7-year-old child with JS	Dynamic neuromuscular stabilization focusing on core stability.	7 months
Ferrão et al., 2025 [19]	Portugal	Case report	9-year-old boy with JS	Multidisciplinary rehabilitation with physiotherapy, occupational therapy, speech therapy and school support.	Long-term follow-up
Mathews et al., 2026 [20]	USA	Case report	18-month-old girl with JS	Multidisciplinary rehabilitation with neurodevelopmental therapy (Bobath concept), trunk and balance training, orthotics, speech and occupational therapy.	3 months + 1-year follow-up
Mański et al., 2026 [21]	Poland	Pilot case series	6 children with genetically confirmed JS (2, 5, 6, 9, 10, 11 years old)	Multidimensional functional assessment including GMFM-88, BARS, musculoskeletal and thoracoabdominal measures.	Single assessment session

Abbreviations: JS, Joubert syndrome; JSRD, Joubert syndrome and related disorders; NDT, neurodevelopmental therapy.

**Table 2:** Rehabilitation approaches reported in the literature and their therapeutic targets.

Rehabilitation Approach	Key Therapeutic Targets	Examples of Interventions Reported in the Literature	Reported Functional Targets
Neurodevelopmental therapy (NDT/Bobath)	Postural control, motor milestone acquisition, trunk stability	Facilitated sitting, weight shifting, transitional movements, developmental positioning	Support trunk stability and sitting balance
Sensory integration therapy	Sensory processing, body awareness, motor planning	Vestibular stimulation, tactile play, proprioceptive activities	Facilitate body awareness and coordination
Core stability/postural control therapy	Central stability, coordination, balance	Dynamic neuromuscular stabilization, trunk activation exercises	Support postural control and functional mobility
Orthotic management	Lower limb alignment, gait stability	Ankle–foot orthoses, knee splints, standing frames, walkers	Support standing ability and assisted gait
Respiratory physiotherapy	Respiratory stability, breathing regulation	Breathing support strategies, airway clearance techniques, respiratory physiotherapy	Support respiratory function and participation in rehabilitation
Multidisciplinary rehabilitation	Global functional development	Combined physical therapy, occupational therapy, speech therapy, family training, educational support	Support functional independence and participation
Oromotor and speech therapy	Feeding, articulation, communication	Oromotor exercises, feeding training, speech–language therapy	Support communication and feeding abilities
Assistive devices and positioning	Postural support, functional activity	Sitting devices, corner seats, adaptive seating systems	Support sitting balance and participation

**Table 3:** Functional outcome measures and reported rehabilitation outcomes in children with Joubert syndrome.

Study	Outcome Measures	Main Findings
Torres et al., 2001 [13]	Battelle Developmental Inventory; Brunet–Lézine Scale	Gradual developmental progress, mainly in cognition and communication
Dekair et al., 2014 [4]	Schedule of Growing Skills	Moderate developmental improvement after rehabilitation
Gagliardi et al., 2015 [14]	Griffiths Scale; WISC; TVPS; VMI; Corsi Block Test; Vineland	Cognitive improvement with persistent visuomotor and executive deficits
İpek et al., 2017 [15]	GMFM-88; WeeFIM	Independent sitting, crawling and walking after intensive physiotherapy
Saiya & Deshpande, 2023 [16]	GMFM-88; Sensory Profile 2	Improvement in gross motor function; independent sitting and walking
Mano et al., 2023 [17]	Enjoji Scale; Vineland-II	Gradual improvement of motor and adaptive skills
Devi et al., 2024 [18]	GMFM-88; WeeFIM	Improved trunk stability and functional independence
Ferrão et al., 2025 [19]	Clinical developmental follow-up	Improved coordination with preserved cognitive abilities
Mathews et al., 2026 [20]	GMFCS; clinical assessment	Improved trunk control, sitting and assisted walking
Mański et al., 2026 [21]	GMFM-88; BARS; musculoskeletal alignment measures; thoracoabdominal configuration and respiratory mobility	Multidimensional functional variability observed; postural control scores did not parallel gross motor performance, suggesting axial control and thoracoabdominal organization as additional functional domains.

Abbreviations: WISC, Wechsler Intelligence Scale for Children; TVPS, Test of Visual Perceptual Skills; VMI, Beery–Buktenica Developmental Test of Visual–Motor Integration; GMFM-88, Gross Motor Function Measure-88; WeeFIM, Functional Independence Measure for Children; GMFCS, Gross Motor Function Classification System.

**Table 4:** Evidence profile of included studies.

Study	Study Type	Sample Size	Key Methodological Limitations
Torres et al., 2001 [13]	Case report	n = 1	Single case; no control group; descriptive design; limited generalisability
Dekair et al., 2014 [4]	Case report	n = 1	Limited intervention detail; no comparator; short follow-up
Gagliardi et al., 2015 [14]	Case report	n = 1	Single case; focus on non-motor domains; limited functional interpretation
İpek et al., 2017 [15]	Case report	n = 1	Intensive therapy; no comparator; limited external validity
Saiya & Deshpande, 2023 [16]	Case report	n = 1	Multiple concurrent interventions; unclear attribution of effects
Mano et al., 2023 [17]	Case series	n = 3	Small heterogeneous sample; variable interventions; no control group
Devi et al., 2024 [18]	Case report	n = 1	Single intervention; short duration; no comparator
Ferrão et al., 2025 [19]	Case report	n = 1	Observational follow-up; lack of standardised outcome measures
Mathews et al., 2026 [20]	Case report	n = 1	Multimodal therapy; no isolation of treatment effects
Mański et al., 2026 [21]	Case series	n = 6	Cross-sectional design; no intervention; exploratory analysis

**Table 5:** Limitations of commonly used pediatric outcome measures in relation to functional domains relevant in Joubert syndrome.

Functional Domain	Relevance in Joubert Syndrome	Representation in Commonly Used Pediatric Outcome Measures	Limitations
Respiratory regulation	Irregular breathing patterns and brainstem dysregulation are common clinical features	Rarely assessed in motor outcome measures	Interaction between respiration and postural control is not captured
Oculomotor and visual function	Oculomotor abnormalities and visual impairment frequently occur	Usually evaluated separately in ophthalmological assessments	Influence on motor organization and balance is not systematically integrated
Sensory integration	Vestibular and proprioceptive processing contributes to balance and coordination	Indirectly reflected through balance tasks	Underlying sensory processing mechanisms are not directly assessed
Cerebellar motor control	Coordination deficits, movement timing disturbances and ataxia are characteristic	Partially reflected in functional task performance	Quality of movement and motor timing are rarely evaluated
Functional motor performance	Mobility, posture and activities of daily living	Captured by GMFM-88 and similar tools	Provides outcome information but limited insight into underlying mechanisms

## Data Availability

No new data were created or analyzed in this study.

## Abbreviations

The following abbreviations are used in this manuscript:

JS	Joubert syndrome
MRI	Magnetic Resonance Imaging
CP	Cerebral Palsy
GMFM-88	Gross Motor Function Measure-88
WeeFIM	Functional Independence Measure for Children
GMFCS	Gross Motor Function Classification System
BARS	Brief Ataxia Rating Scale
NDT	Neurodevelopmental Therapy
DNS	Dynamic Neuromuscular Stabilization
JSRD	Joubert Syndrome and Related Disorders
BDI	Battelle Developmental Inventory
SGS	Schedule of Growing Skills
WISC	Wechsler Intelligence Scale for Children
TVPS	Test of Visual Perceptual Skills
VMI	Beery–Buktenica Developmental Test of Visual–Motor Integration
ICF	International Classification of Functioning, Disability and Health
